# Comparing dietary strategies to manage cardiovascular risk in primary care: a narrative review of systematic reviews

**DOI:** 10.3399/BJGP.2022.0564

**Published:** 2024-02-20

**Authors:** Hannah Greenwood, Katelyn Barnes, Lauren Ball, Paul Glasziou

**Affiliations:** Institute for Evidence-Based Healthcare, Faculty of Health Science & Medicine, Bond University, Gold Coast.; Centre for Community Health and Wellbeing, University of Queensland, Brisbane; senior research officer, Academic Unit of General Practice, ACT Health Directorate; School of Medicine and Psychology, The Australian National University, Canberra.; Centre for Community Health and Wellbeing, University of Queensland, Brisbane.; Institute for Evidence-Based Healthcare, Faculty of Health Science & Medicine, Bond University, Gold Coast.

**Keywords:** cardiovascular diseases, general practice, lifestyle, nutritional sciences, primary health care, review

## Abstract

**Background:**

Nutrition care in general practice is crucial for cardiovascular disease (CVD) prevention and management, although comparison between dietary strategies is lacking.

**Aim:**

To compare the best available (most recent, relevant, and high-quality) evidence for six dietary strategies that are effective for primary prevention/absolute risk reduction of CVD.

**Design and setting:**

A pragmatic narrative review of systematic reviews of randomised trials focused on primary prevention of cardiovascular events.

**Method:**

Studies about: 1) adults without a history of cardiovascular events; 2) target dietary strategies postulated to reduce CVD risk; and 3) direct cardiovascular or all-cause mortality outcomes were included. Six dietary strategies were examined: energy deficit, Mediterranean-like diet, sodium reduction (salt reduction and substitution), the Dietary Approaches to Stop Hypertension (DASH) diet, alcohol reduction, and fish/fish oil consumption. Reviews were selected based on quality, recency, and relevance. Quality and certainty of evidence was assessed using GRADE.

**Results:**

Twenty-five reviews met inclusion criteria; eight were selected as the highest quality, recent, and relevant. Three dietary strategies showed modest, significant reductions in cardiovascular events: energy deficit (relative risk reduction [RRR] 30%, 95% confidence interval [CI] = 13 to 43), Mediterranean-like diet (RRR 40%, 95% CI = 20 to 55), and salt substitution (RRR 30%, 95% CI = 7 to 48). Still, some caveats remain on the effectiveness of these dietary strategies. Salt reduction, DASH diet, and alcohol reduction showed small, significant reductions in blood pressure, but no reduction in cardiovascular events. Fish/fish oil consumption showed little or no effect; supplementation of fish oil alone showed small reductions in CVD events.

**Conclusion:**

For primary prevention, energy deficit, Mediterranean-like diets, and sodium substitution have modest evidence for risk reduction of CVD events. Strategies incorporated into clinical nutrition care should ensure guidance is person centred and tailored to clinical circumstances.

## Introduction

The World Health Organization estimates that 75% of cardiovascular events may be preventable. However, cardiovascular disease (CVD) is a major source of morbidity and mortality globally.^1^ Appropriate primary care management of absolute CVD risk reduces associated death and disability,^2^ and contemporary CVD risk management focuses on reducing absolute risk (that is, 5-year risk of CVD including multiple risk factors), rather than individual risk factors.^3^^,^^4^

Diet is a key factor in managing absolute CVD risk,^4^ and is recognised for the primary prevention of CVD,^1^^,^^5^^,^^6^ but the relative effectiveness of different dietary strategies remains unclear. Direct comparison of different dietary strategies will support clinicians to make evidence-informed diet recommendations for patients looking to manage CVD risk. Although other dietary approaches (for example, vegetarian or vegan diets)^7^ may influence CVD risk, this narrative review of systematic reviews focuses on six strategies identified as potentially influencing absolute cardiovascular risk by the National Heart Foundation of Australia and an expert advisory panel.^8^^,^^9^ These are outlined in Box 1 along with their proposed cardiovascular effects. Given the heterogeneous nature of these interventions, a narrative review was conducted to allow top-level comparison between dietary strategies to inform clinical decisions.

**Box 1.** Target dietary strategies and their cardiovascular effects

**Dietary strategy**	**Description of dietary strategy**	**Cardiovascular effects**
**Energy deficit**	Diets specifically formulated to reduce calorie intake (for example, very-low energy diets [<800 kcal/day]) or lifestyle change to induce an energy deficit (including diet with or without exercise)	Decreased body weight lowers blood pressure^10^

**Mediterranean diet**	A naturalistic dietary pattern that promotes high intakes of wholegrains, vegetables, and fruits, moderate intakes of seafood, unsaturated fats, and red wine, and limited intake of red meats. Regular exercise is promoted as part of the lifestyle	Combination of lifestyle factors thought to be cardioprotective via reduced blood pressure, and reduced blood lipids, among other mechanisms^11^

**Sodium reduction**		
Salt reduction	Table salt (NaCl) intake is decreased through reduction of added table salt to foods, or manipulation of table salt intake to allow for comparison between higher and lower salt intake groups	Decreased sodium lowers blood pressure^12^
Salt substitution	Sodium in regular table salt or other high sodium products is replaced with potassium (KCl)	Decreased sodium lowers blood pressure;^12^ increased potassium may have cardioprotective effects^13^

**DASH diet**	Dietary pattern designed specifically to reduce hypertension that promotes high intakes of fruits and vegetables, moderate intake of wholegrains and low-fat dairy, moderate-to-limited intakes of meats, and limited intake of fats and salt	Combination of lifestyle factors thought to reduce blood pressure^14^

**Alcohol reduction**	Reduction in usual alcohol intake or elimination of alcohol from diet	Decreased alcohol is thought to lower blood pressure. Alcohol consumption has a complex relationship with cardiovascular health and excess consumption is associated with many CV diseases, so reduction may reduce CV disease risk^15^^,^^16^

**Fish/fish oil consumption**	Diets high in fish, or supplemental fish oils	Omega chain fatty acids, commonly found in fish, are thought to be cardioprotective^17^

*CV = cardiovascular. DASH = Dietary Approaches to Stop Hypertension.*

In this pragmatic narrative review of systematic reviews, the aim was to identify and descriptively compare the most relevant, best available, highest-quality systematic reviews for six dietary strategies postulated to be effective for primary prevention/absolute risk reduction of CVD:

- energy deficit;
- Mediterranean-like diet;
- sodium reduction (salt reduction and substitution);
- Dietary Approaches to Stop Hypertension (DASH) diet;
- alcohol reduction; and
- fish/fish oil consumption.^8^

**Table:** How this fits in

Diet is a key factor in preventing cardiovascular disease (CVD) and managing absolute CVD risk, but the comparative effectiveness of different dietary strategies to reduce absolute CVD risk is unclear. By examining current best available evidence this study found that energy reduction, Mediterranean-style diets, and salt substitution are the most promising to reduce CVD events, although all the examined strategies can help absolute CVD risk reduction. Using behaviour change principles, clinicians can work with patients to select the dietary strategy/ies most aligned with their specific personal and clinical circumstances.

## Method

This narrative review has been reported in line with the Preferred Reporting Items for Systematic Reviews and Meta-Analyses (PRISMA) guidelines.^18^ A broad methodological approach was prospectively developed as part of a larger programme of commissioned work to update Australian absolute CVD risk guidelines. As a result of this, the protocol was not registered. Pragmatic decisions were made to enable identification of the most recent, relevant, and high-quality available evidence. In brief, studies published after 2014 were included to limit screening and identify the most recent evidence; the review was limited to six dietary strategies in line with the funder’s priorities;^8^^,^^9^ it utilised a targeted search approach (that is, a two-pronged approach) to identify included reviews; and used AMSTAR 1 rather than AMSTAR 2 to assess quality as it allows for an overall score. These decisions and full justifications are summarised in Supplementary Information S1.

### Identification of target dietary interventions

Target dietary interventions (outlined in Box 1) relevant to clinical practice were identified jointly by general practice, cardiovascular and nutrition and dietetics experts from the authorship team, their networks, and the National Heart Foundation cardiovascular expert committee.^8^

### Search strategy

A two-pronged citation analysis approach was used to identify reviews for each dietary strategy.^19^ First, ‘similar articles’ searches in PubMed were conducted from known seed articles.^20^ Second, for each review identified, a forward and backward citation analysis was conducted using the SpiderCite automation tool,^21^ and results were filtered to include only systematic reviews.

### Screening and study selection

Systematic reviews of randomised controlled trials (RCTs) published from January 2014 to May 2022 were screened for eligibility against PICO (population, intervention, comparator, outcome) criteria in Box 2. If a review for a target diet reporting direct cardiovascular or mortality outcomes was not found, reviews with indirect outcomes (for example, blood pressure and blood lipids) were considered.

**Box 2.** PICO inclusion and exclusion criteria

**Inclusion**	**Exclusion**
**Population:** General population/primary prevention cohort (including hypertension) of any age, if adults are included	**Population:** Secondary prevention cohort (participants had existing CVD)
**Intervention:** Dietary non-drug interventions (energy deficit, Mediterranean diet, sodium reduction [salt reduction or substitution], DASH diet, alcohol reduction, and fish/fish oil consumption)	**Intervention:** Other diet intervention not pre-specified by expert advisors
**Comparator:** Any other intervention or control	**Comparator:** None
**Outcome:** CVD event or all-cause mortality	**Outcome:** Direct nor indirect cardiovascular outcomes reported

*CVD = cardiovascular disease. DASH = Dietary Approaches to Stop Hypertension. PICO = population, intervention, comparator, outcome.*

At prong 1, one reviewer (the first author) screened systematic reviews for those relevant to the PICO of each target dietary intervention. At prong 2, two reviewers (the first author and the second or third author) independently screened title and abstract to select eligible reviews, including those identified in prong 1. The full text was obtained and was independently assessed for eligibility and risk of bias by two reviewers (the first author and the second or third author). Discrepancies in screening were resolved by discussion or referral to another author (the senior author).

Selection of key systematic review/s for each dietary intervention was made jointly by all authors, accounting for recency, quality, and relevance (that is, how closely the review question matched the PICO of the six selected dietary approaches). If the outcomes in a review did not include both indirect (for example, blood pressure) and direct (for example, CVD events or mortality) measures of cardiovascular risk, the authors considered and included more than one review to summarise findings where appropriate. If studies were related and included additional information (for example, subsequent trials or reanalysis), these results are also reported in the current article.

### Review methodological quality (risk of bias)

AMSTAR 1 was used to assess methodological quality of systematic reviews across 11 domains, and it is considered a pragmatic, time-efficient method for comparing quality between studies.^22^ Two authors (the first author and the second or third author) independently assessed risk of bias for all full-text results.

### Data extraction

Where available, the following outcome data were extracted: dietary strategy, number of included trials and participants, participant hypertension status (hypertensive, mixed), intervention, length of intervention, adherence to intervention, comparator, all relevant outcomes as reported (that is, total [all-cause] mortality), CVD mortality, CVD events, blood pressure (systolic, diastolic), body weight, blood lipid concentration, change in alcohol consumption, length of study follow-up, and effect estimate (hazard ratio [HR] or risk ratio [RR]) with 95% confidence intervals (CIs) and/or *P*-values.

### Evaluation of evidence certainty

The certainty or quality of the body of evidence for each reported outcome was assessed using GRADE.^23^ GRADE provides a rating reflecting how certain the authors are that estimated effect aligns with the true effect: very low, low, moderate, or high. Where original authors had conducted GRADE, results were reviewed and retained if sufficiently detailed and applicable. For reviews without existing GRADE assessment, two authors (the first and second authors) completed GRADE independently, with disputes resolved by discussion or referral to another author (the third or senior author).

### Data synthesis

Existing outcomes for the dietary strategies were tabulated to allow direct descriptive comparison between strategies. A narrative overview of findings compares the different strategies for primary CVD prevention. No new statistical analyses were planned because of the heterogeneity of interventions, timeframes, review questions, and populations.

## Results

**Figure 1.**
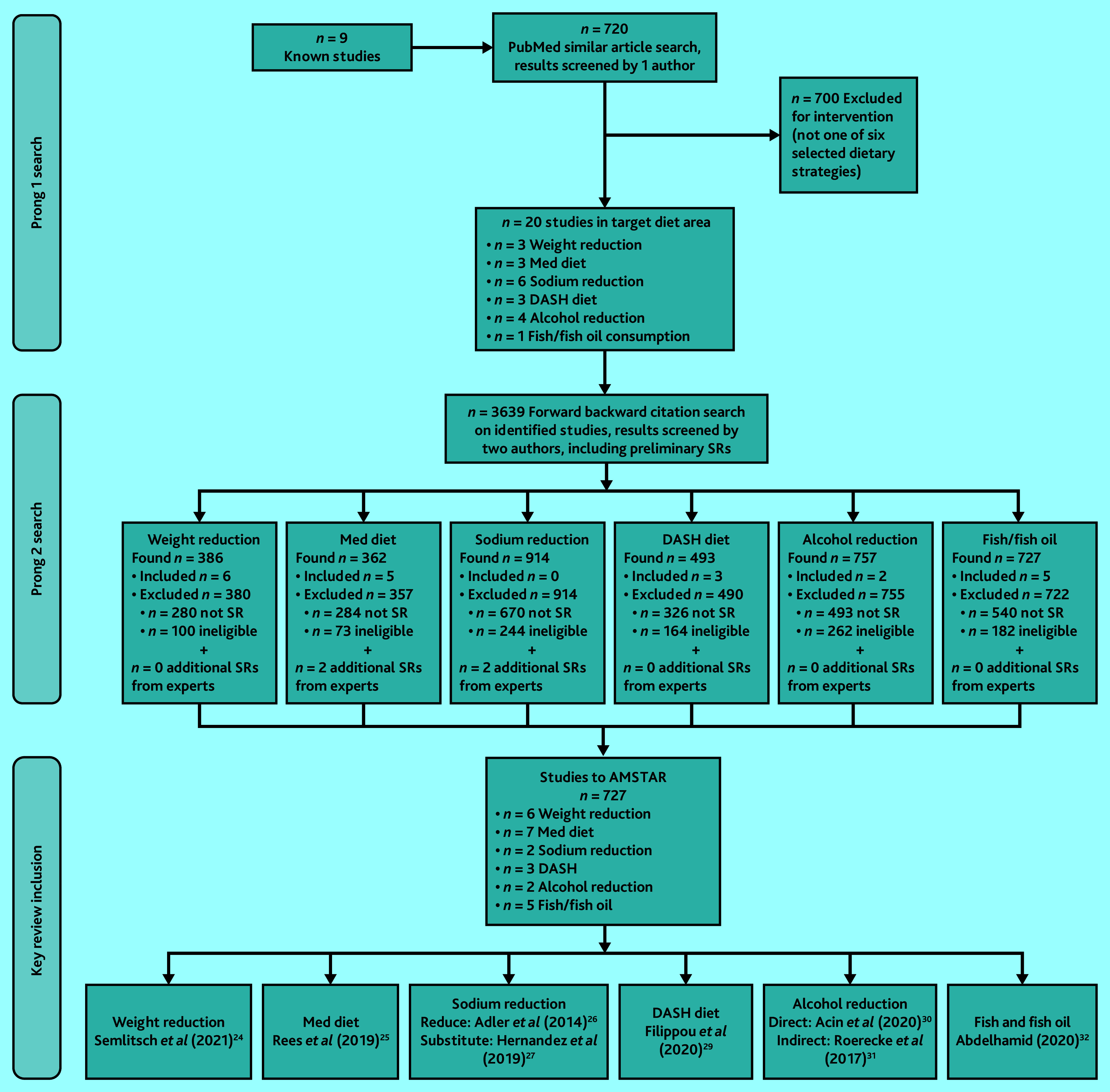
Preferred Reporting Items for Systematic Reviews and Meta-Analyses (PRISMA) study flow diagram to select key reviews. DASH = Dietary Approaches to Stop Hypertension. Med = Mediterranean. SR = systematic review.

The selection process is presented in Figure 1. Eight reviews were included for the six dietary strategies. Supplementary Table S1 describes each included review, including interventions.

**Figure 2.**
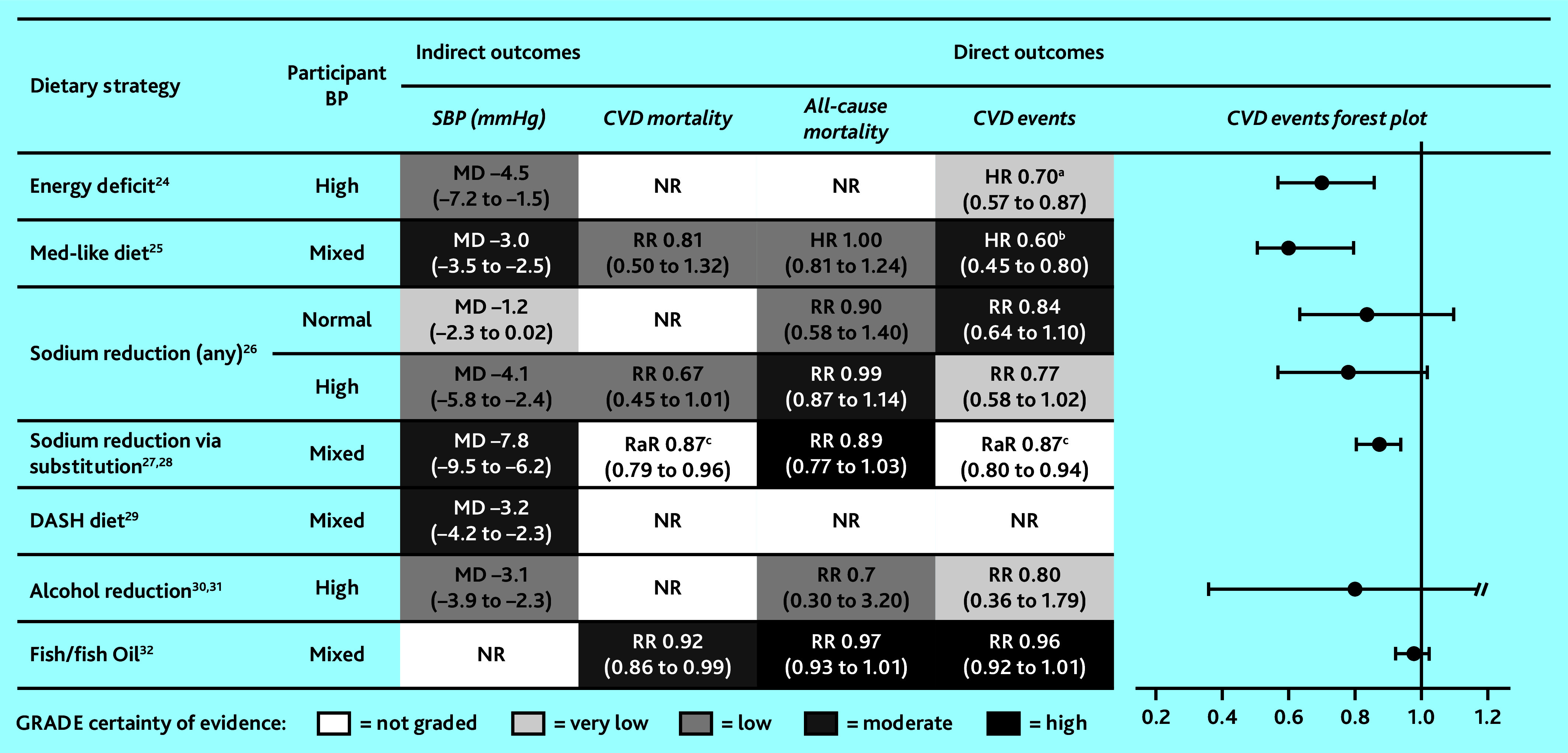
Summary of key cardiovascular outcomes for target dietary strategies, including forest plot of HR/RaR/RR and associated 95% CIs to visually display effect of dietary strategies on CVD event outcomes. ^a^Combined endpoint including CVD complications. ^b^Stroke only. ^c^NR in systematic review; results of subsequent randomised controlled trial, Neal *et al* (2021).^28^ BP = blood pressure. CVD = cardiovascular disease. DASH = Dietary Approaches to Stop Hypertension. DBP = diastolic blood pressure. HR = hazard ratio. MD = mean difference. Med = Mediterranean. NR = not reported. RaR = rate ratio. RR = risk ratio. SBP = systolic blood pressure.

Figure 2 provides a summary of key direct and indirect cardiovascular outcomes alongside certainty of evidence. See Supplementary Information S2 for new GRADE assessments and summaries of existing GRADE assessments, and Supplementary Table S2 for description and AMSTAR quality rating of all studies assessed at full text, and reasons for inclusion/exclusion.

### Energy deficit

Semlitsch *et al* (2021)^24^ reported low-certainty evidence that energy deficit strategies (with or without exercise) lead to modest reductions in blood pressure (mean difference [MD] −4.5 mmHg, 95% CI = −7.2 to −1.5) among people with hypertension who were overweight, but had clinically meaningful weight loss compared with controls (MD −3.98 kg, 95% CI = −4.79 to −3.17). One included trial found energy deficit meaningfully lowered risk of CVD events (HR 0.70, 95% CI = 0.57 to 0.87), but evidence certainty was low. No evidence was available regarding mortality.

### Mediterranean diet

Rees *et al* (2019)^25^ reported that, compared with low-fat diets, there is moderate-certainty evidence that Mediterranean-like diets meaningfully reduce strokes (HR 0.60, 95% CI = 0.45 to 0.80). Evidence is inconclusive for CVD mortality: effect estimates show reduced risk but are imprecise with wide CIs (RR 0.81, 95% CI = 0.50 to 1.32). There is little or no evidence of effect of Mediterranean-like diet on all-cause mortality (HR 1.00, 95% CI = 0.81 to 1.24). Moderate-certainty evidence suggests that, compared with no/minimal intervention, Mediterranean-like diets had a small but meaningful effect on blood pressure (systolic blood pressure [SBP] MD −3.0 mmHg, 95% CI = −3.5 to −2.5; diastolic blood pressure [DBP] MD −2.0 mmHg, 95% CI = −2.3 to −1.7), but little to no effect on blood lipids (low certainty; low density lipoprotein [LDL]: MD −0.08 mmol/L, 95% CI = −0.26 to 0.09; high-density lipoprotein [HDL]: MD 0.02 mmol/L, 95% CI = −0.04 to 0.08). Compared with other dietary interventions, Mediterranean-like diet had little to no clear effect on blood pressure or lipids.

### Sodium reduction

Adler *et al* (2014)^26^ reported that sodium reduction either through salt reduction or substitution of <70–100 mmol/day for people with hypertension appeared to reduce blood pressure (SBP MD −4.1 mmHg, 95% CI = −5.8 to −2.4; DBP MD −3.7 mmHg, 95% CI = −8.4 to 0.93), CVD mortality (RR 0.67, 95% CI = 0.45 to 1.01), and CVD events (RR 0.77, 95% CI = 0.58 to 1.02). Although CIs are uncertain and evidence certainty is low, the risk reductions are clinically meaningful.

For people with normotensive blood pressure, there is moderate-to-high certainty evidence that sodium reduction does not meaningfully reduce CVD events (RR 0.84, 95% CI = 0.64 to 1.10) or all-cause mortality (RR 0.90, 95% CI = 0.58 to 1.40). These findings are driven by one trial assessing large-scale salt reduction by institutional low-sodium alternative substitution (salt substitution).

### Salt substitution

Hernandez *et al* (2019)^27^ reported high-certainty evidence that salt substitution alone meaningfully reduced all-cause mortality (RR 0.89, 95% CI = 0.77 to 1.03), although CIs remain inconclusive. In the mixed hyper- and normotensive sample, salt substitution meaningfully reduced SBP (MD −7.8 mmHg, 95% CI = −9.5 to −6.2), although diastolic reductions were more modest (MD −3.96 mmHg; 95% CI = −5.17 to −2.74).

Although no meta-analysis of CVD mortality or events were reported, a large subsequent randomised trial of salt substitution (75% sodium chloride, 25% potassium chloride) versus regular salt found significant and clinically important reductions in SBP, stroke (rate ratio 0.86, 95% CI = 0.77 to 0.96), major CVD events (rate ratio 0.87, 95% CI = 0.80 to 0.94), and death (rate ratio 0.87, 95% CI = 0.79 to 0.96).^28^ Importantly, adverse events from high potassium were not significantly higher in the substitution group (rate ratio 1.04, 95% CI = 0.80 to 1.37).

### DASH diet

Filippou *et al* (2020)^29^ reported moderate-certainty evidence that, over an average of 15.3 weeks, the DASH diet (compared with a control diet) had a small effect on blood pressure reduction (SBP MD −3.2 mmHg, 95% CI = −4.2 to −2.3; DBP MD −2.5 mmHg, 95% CI = −3.5 to −1.5); this was consistent regardless of baseline blood pressure. The blood pressure lowering effect of the DASH diet was more pronounced when participants’ baseline sodium intake was >2400 mg/day (*P* = 0.003), and when participants were aged <50 years (*P*<0.001). There was no moderating effect of weight, and no evidence was reported for the impact of DASH diet for primary prevention of cardiovascular events or mortality.

### Alcohol reduction

Acin *et al* (2020)^30^ reported little to no effect of alcohol reduction on all-cause mortality (RR 0.70, 95% CI = 0.20 to 3.20) or cardiovascular events (RR 0.80, 95% CI = 0.36 to 1.79), but evidence was of low certainty and drawn from a subgroup of one trial with mostly male participants. No included studies reported CVD mortality or blood pressure. Roerecke *et al* (2017)^31^ reported low-certainty evidence that reduced alcohol consumption meaningfully decreased blood pressure (SBP MD −3.1 mmHg, 95% CI = −3.9 to −2.3; DBP MD −2.0 mmHg, 95% CI = −2.7 to −1.4). Substantial heterogeneity was largely explained when stratified by alcohol consumption at baseline. When consuming ≤2 drinks/day at baseline, reduction had no significant effect on blood pressure, whereas for those consuming ≥3 drinks/day at baseline alcohol reduction resulted in meaningful reductions in blood pressure. This effect was strongest for those consuming ≥6 drinks/day at baseline (SBP MD −5.5 mmHg, 95% CI = −6.7 to −4.3).

### Fish/fish oil

Abdelhamid (2020)^32^ reported high-certainty evidence that fish or fish oil had a very small effect on CVD mortality (RR 0.92, 95% CI = 0.86 to 0.99), but little to no effect on all-cause mortality (RR 0.97, 95% CI = 0.93 to 1.01), CVD events (RR 0.96, 95% CI = 0.92 to 1.01), or cholesterol (LDL: MD 0.01 mmol/L, 95% CI = −0.01 to 0.03; HDL: MD 0.03 mmol/L, 95% CI = 0.01 to 0.05). There was low-certainty evidence of a small reduction in coronary heart disease (CHD) mortality (RR 0.90, 95% CI = 0.81 to 1.00) and CHD events (RR 0.91, 95% CI = 0.85 to 0.97), but numbers needed to treat were high. Bernasconi *et al*’s (2020)^33^ reanalysis of supplementation alone (average dose 1221 mg/day) found reduced risk of CHD events, fatal and non-fatal myocardial infarction, and CHD mortality, but not CVD events. Some outcomes were dose dependent, whereby higher doses of fish oil increased protection from CVD events and myocardial infarction. Although there was no clear evidence for an impact on CVD of including fish in the diet, when supplementation alone was considered, there was a small, dose-dependent, protective cardiovascular effect of fish oil supplementation, although number needed to treat for additional benefit was high.^32^^,^^33^

## Discussion

### Summary

This pragmatic narrative review of systematic reviews descriptively compares the effectiveness of six dietary strategies on cardiovascular outcomes in primary prevention populations. Although no singular dietary strategy produced significant risk reductions across all CVD risk or mortality outcomes, all dietary strategies showed some potential benefit to meaningfully reduce CVD events and improve indirect CVD outcomes (for example, blood pressure), overall reducing CVD risk; however, there are some caveats to note within and across dietary strategies.

Three dietary strategies showed modest, significant reductions in cardiovascular events: energy deficit (relative risk reduction [RRR] 30%, 95% confidence interval [CI] = 13 to 43), Mediterranean-like diet (RRR 40%, 95% CI = 20 to 55), and salt substitution (RRR 30%, 95% CI = 7 to 48). The evidence certainty for energy deficit was very low, and the composite outcome used (cardiovascular complications + recommencing antihypertensives) may inflate the estimate.^24^ Mediterranean-like diets compared with low-fat diets showed hazard reduction, but this was for stroke only, and from one large study (reanalysis of PREDIMED). The salt substitution effect estimate is from a large trial^28^ and is not a pooled estimate. Although the point estimates for sodium reduction (via salt reduction or salt substitution), alcohol reduction, or fish/fish oil also suggest reduced CVD event risk, the CIs were too imprecise to confirm or exclude important differences.

Most strategies that reported blood pressure showed small but meaningful reductions in SBP.^24^^,^^25^^,^^27^^,^^31^^,^^34^ The most promising strategy reviewed is salt substitution, a type of sodium-reduction strategy.^27^ The reported SBP reduction (6 mmHg to 9 mmHg) is sufficient to reduce CVD mortality, based on estimates that systolic reductions of 1 mmHg and 4 mmHg translate to reductions in CVD mortality of 2% and 8%, respectively.^35^

Mortality outcomes were not available for all dietary strategies. For CVD mortality, salt substitution showed a significant reduction in CVD mortality, although this estimate is from a large trial, not a meta-analysis.^28^ Other available point estimates show small reductions in CVD mortality risk, but CIs are too imprecise to determine if these are meaningful. For all-cause mortality, although available point estimates show a small risk reduction, no strategy was associated with significantly reduced all-cause mortality.

Some caveats across all dietary approaches should be highlighted. First, the length of the studies in each of the included reviews are arguably not long enough to capture lifelong prevention of CVD, and the distinction between intervention and follow-up can be ambiguous because of the assumption of permanent dietary change.^36^ Second, very few studies within the included reviews reported adherence to the dietary intervention or assessed dietary intake. Dietary intake is an important indication of intervention fidelity and is required to differentiate a dietary intervention’s CVD risk-reduction effects compared with other physiological effects. Finally, very few RCTs reported outcome measures that reflect potential mechanisms of action of the dietary intervention, for example, whether energy deficit lowers CVD event risk via indirect lowering of blood pressure or via direct mechanisms.

### Strengths and limitations

First, in the current study the authors elected to take a pragmatic approach to identify the most recent, highest-quality reviews for a selection of six relevant dietary strategies, with the goal to approximate effect sizes of each dietary strategy using best available evidence, not all available evidence. However, it is recognised that this approach does not provide a definitive measure of effect for each dietary strategy considering all available evidence, which would be a substantially larger, more resource-intensive project.

Second, the six dietary strategies were pragmatically selected by an expert committee for being relevant to clinical practice and postulated to effectively reduce absolute cardiovascular risk. However, it is highlighted that this is not an exhaustive list of dietary strategies that may reduce cardiovascular risk. Other examples of dietary strategies touted for beneficial cardiovascular effects that were outside the scope of this work include low carbohydrate and intermittent fasting, although recent Cochrane reviews suggest no major cardiovascular benefit.^37^^,^^38^

Third, only reviews of RCTs were examined because of quality limitations inherent to observational studies. However, RCT designs for dietary studies also have limitations, such as difficulties with blinding, control of intervention fidelity, long-term follow-up, uniformity, independence of effects, and control over comparator groups.^39^^,^^40^ Assessing long-term outcomes is required to understand the impact of diet on cardiovascular health, but dietary interventions are challenging to implement long term, particularly using RCT designs.

Fourth, as this is a narrative review of heterogeneous interventions, meta-analysis and subgroup analysis were precluded. Although conclusions cannot be drawn for specific population subgroups, this review allows for top-level comparison between different dietary strategies to enable evidence-informed clinical decisions for recommending dietary strategies to manage cardiovascular risk in primary prevention populations.

Finally, there may be some overlap between dietary strategies, for example, the Mediterranean diet may be used as an energy deficit strategy. However, the authors of the current study suggest there is a distinction between dietary strategies with the primary aim of reducing weight via energy deficit (such as the reduced-calorie diets in the included energy deficit study)^24^ and approaches such as the Mediterranean diet or DASH that may incidentally result in weight loss but this is not the primary aim. Although this justifies the authors’ inclusion of both dietary strategies, it does not erase possible overlapping effects that should be considered in interpretation of findings.

### Comparison with existing literature

Given the social and economic burden of CVD, and the central role diet plays in the prevention and management of CVD,^1^^,^^5^^,^^6^ consideration of how to support primary care patients to optimise their diet is essential. To provide positive, cost-effective, and sustainable dietary change, current evidence supports individual, community, and system-level strategies.^41^^,^^42^ The complexity of dietary interventions examined in this narrative review varied from simple behaviour change (for example, salt substitution that requires only direct replacement of regular salt with salt substitute) to more complex behaviour change (for example, Mediterranean or DASH diets that require adopting a comprehensive lifestyle approach). Dietary interventions that use behaviour change science are likely to better facilitate improvements in health outcomes.^43^

The selected reviews rarely considered the complexity of behaviour change required to successfully implement different dietary strategies, and clinicians should be guided by behaviour change theories when supporting patients to make dietary changes. There is inherent complexity in the variability of dietary behaviours, which are highly individual and influenced by personal (for example, taste and food preference), social (for example, familial/cultural preferences), and environmental factors (for example, ability to obtain, store, prepare, and cook foods appropriately).^44^ For GPs exploring diet change for cardiovascular health with patients, selecting dietary strategies to match their patients’ needs, preferences, access, and social determinants of behaviour may maximise person-centred improvements in diet and health outcomes.^45^

Nutrition care is not provided as often as clinically beneficial, and patients expect primary care clinicians to be competent in supporting them to optimise their diet.^46^ This narrative review shows that several dietary approaches are protective for cardiovascular health, so improving primary care clinicians’ skills to support patient dietary change is worth pursuing. Clinicians can also advocate for positive nutrition policies at the community and system levels.^47^ This may include identifying misinformation, promoting the relevance and importance of healthy eating, and linking patients to evidence-based reputable sources of further support, including referral to a registered or accredited dietitian, which is shown to be clinically effective for reducing blood lipid levels.^48^^,^^49^

### Implications for practice

All dietary interventions reviewed showed promising but modest effects on direct or indirect CVD outcomes and may be recommended by GPs for cardiovascular risk reduction, although, as discussed, there are some caveats to consider. The choice of dietary strategy for patients will depend on preferences and circumstances that may enable sustained behaviour change. Assessments of current weight (for energy deficit strategy), sodium intake (for salt reduction or substitution in people with hypertension), and alcohol intake (for high alcohol intake) should be conducted, and a Mediterranean diet may be useful whatever the background factors. Crucially, dietary strategies are not mutually exclusive: multiple compatible strategies (for example, sodium substitution and alcohol reduction) may be applied simultaneously to potentially maximise the benefit to cardiovascular health and absolute risk reduction.

## References

[1] World Health Organization Cardiovascular diseases. https://www.who.int/health-topics/cardiovascular-diseases#tab=tab_1.

[2] Albarqouni L, Doust JA, Magliano D (2019). External validation and comparison of four cardiovascular risk prediction models with data from the Australian Diabetes, Obesity and Lifestyle study. Med J Aust.

[3] Jackson R, Lawes CM, Bennett DA (2005). Treatment with drugs to lower blood pressure and blood cholesterol based on an individual’s absolute cardiovascular risk. Lancet.

[4] National Vascular Disease Prevention Alliance (2012). Guidelines for the management of absolute cardiovascular disease risk.

[5] Arnett DK, Blumenthal RS, Albert MA (2019). 2019 ACC/AHA guideline on the primary prevention of cardiovascular disease: a report of the American College of Cardiology/American Heart Association Task Force on Clinical Practice Guidelines. J Am Coll Cardiol.

[6] Visseren FLJ, Mach F, Smulders YM (2021). 2021 ESC Guidelines on cardiovascular disease prevention in clinical practice: developed by the task force for cardiovascular disease prevention in clinical practice with representatives of the European Society of Cardiology and 12 medical societies with the special contribution of the European Association of Preventive Cardiology (EAPC). Eur Heart J.

[7] Dybvik JS, Svendsen M, Aune D (2023). Vegetarian and vegan diets and the risk of cardiovascular disease, ischemic heart disease and stroke: a systematic review and meta-analysis of prospective cohort studies. Eur J Nutr.

[8] Institute for Evidence-Based Healthcare (2021). Evidence synthesis to support the development of guidelines for absolute cardiovascular disease risk.

[9] Heart Foundation. Evidence Synthesis Report.

[10] Lavie CJ, Milani RV, Ventura HO (2009). Obesity and cardiovascular disease: risk factor, paradox, and impact of weight loss. J Am Coll Cardiol.

[11] Schwingshackl L, Morze J, Hoffmann G (2020). Mediterranean diet and health status: active ingredients and pharmacological mechanisms. Br J Pharmacol.

[12] He FJ, Tan M, Ma Y, MacGregor GA (2020). Salt reduction to prevent hypertension and cardiovascular disease: JACC state-of-the-art review. J Am Coll Cardiol.

[13] Greer RC, Marklund M, Anderson CAM (2020). Potassium-enriched salt substitutes as a means to lower blood pressure: benefits and risks. Hypertension.

[14] Lin P-H, Allen JD, Li Y-J (2012). Blood pressure-lowering mechanisms of the DASH dietary pattern. J Nutr Metab.

[15] Di Castelnuovo A, Costanzo S, di Giuseppe R (2009). Alcohol consumption and cardiovascular risk: mechanisms of action and epidemiologic perspectives. Future Cardiol.

[16] Piano MR (2017). Alcohol’s effects on the cardiovascular system. Alcohol Res.

[17] Endo J, Arita M (2016). Cardioprotective mechanism of omega-3 polyunsaturated fatty acids. J Cardiol.

[18] Moher D, Liberati A, Tetzlaff J, Altman DG (2009). Preferred Reporting Items for Systematic Reviews and Meta-Analyses: the PRISMA statement. BMJ.

[19] Robinson KA, Dunn AG, Tsafnat G, Glasziou P (2014). Citation networks of related trials are often disconnected: implications for bidirectional citation searches. J Clin Epidemiol.

[20] Sampson M, de Bruijn B, Urquhart C, Shojania K (2016). Complementary approaches to searching MEDLINE may be sufficient for updating systematic reviews. J Clin Epidemiol.

[21] Clark J, Glasziou P, Del Mar C (2020). A full systematic review was completed in 2 weeks using automation tools: a case study. J Clin Epidemiol.

[22] Shea BJ, Grimshaw JM, Wells GA (2007). Development of AMSTAR: a measurement tool to assess the methodological quality of systematic reviews. BMC Med Res Methodol.

[23] Schünemann H, Brożek J, Guyatt G, Oxman A (2013). GRADE Handbook.

[24] Semlitsch T, Krenn C, Jeitler K (2021). Long-term effects of weight-reducing diets in people with hypertension. Cochrane Database Syst Rev.

[25] Rees K, Takeda A, Martin N (2019). Mediterranean-style diet for the primary and secondary prevention of cardiovascular disease. Cochrane Database Syst Rev.

[26] Adler AJ, Taylor F, Martin N (2014). Reduced dietary salt for the prevention of cardiovascular disease. Cochrane Database Syst Rev.

[27] Hernandez AV, Emonds EE, Chen BA (2019). Effect of low-sodium salt substitutes on blood pressure, detected hypertension, stroke and mortality: a systematic review and meta-analysis of randomised controlled trials. Heart.

[28] Neal B, Wu Y, Feng X (2021). Effect of salt substitution on cardiovascular events and death. N Engl J Med.

[29] Filippou CD, Tsioufis CP, Thomopoulos CG (2020). Dietary Approaches to Stop Hypertension (DASH) diet and blood pressure reduction in adults with and without hypertension: a systematic review and meta-analysis of randomized controlled trials. Adv Nutr.

[30] Acin MT, Rueda JR, Saiz LC (2020). Alcohol intake reduction for controlling hypertension. Cochrane Database Syst Rev.

[31] Roerecke M, Kaczorowski J, Tobe SW (2017). The effect of a reduction in alcohol consumption on blood pressure: a systematic review and meta-analysis. Lancet Public Health.

[32] Abdelhamid AS, Brown TJ, Brainard JS (2020). Omega-3 fatty acids for the primary and secondary prevention of cardiovascular disease. Cochrane Database Syst Rev.

[33] Bernasconi AA, Wiest MM, Lavie C (2021). Effect of omega-3 dosage on cardiovascular outcomes: an updated meta-analysis and meta-regression of interventional trials. Mayo Clin Proc.

[34] Filippou CD, Thomopoulos CG, Kouremeti MM (2021). Mediterranean diet and blood pressure reduction in adults with and without hypertension: a systematic review and meta-analysis of randomized controlled trials. Clin Nutr.

[35] MacMahon S, Peto R, Collins R (1990). Blood pressure, stroke, and coronary heart disease: part 1, prolonged differences in blood pressure: prospective observational studies corrected for the regression dilution bias. Lancet.

[36] Koehler K, Drenowatz C (2019). Integrated role of nutrition and physical activity for lifelong health. Nutrients.

[37] Allaf M, Elghazaly H, Mohamed OG (2021). Intermittent fasting for the prevention of cardiovascular disease. Cochrane Database Syst Rev.

[38] Naude CE, Brand A, Schoonees A (2022). Low-carbohydrate versus balanced-carbohydrate diets for reducing weight and cardiovascular risk. Cochrane Database Syst Rev.

[39] Schwingshackl L, Schünemann HJ, Meerpohl JJ (2021). Improving the trustworthiness of findings from nutrition evidence syntheses: assessing risk of bias and rating the certainty of evidence. Eur J Nutr.

[40] Zeilstra D, Younes JA, Brummer RJ, Kleerebezem M (2018). Perspective: fundamental limitations of the randomized controlled trial method in nutritional research: the example of probiotics. Adv Nutr.

[41] Ananthapavan J, Sacks G, Brown V (2020). Priority-setting for obesity prevention — the Assessing Cost-Effectiveness of obesity prevention policies in Australia (ACE-Obesity Policy) study. PLoS One.

[42] Vincze L, Barnes K, Somerville M (2021). Cultural adaptation of health interventions including a nutrition component in Indigenous peoples: a systematic scoping review. Int J Equity Health.

[43] Rigby RR, Mitchell LJ, Hamilton K, Williams LT (2020). The use of behavior change theories in dietetics practice in primary health care: a systematic review of randomized controlled trials. J Acad Nutr Diet.

[44] Swan WI, Vivanti A, Hakel-Smith NA (2017). Nutrition care process and model update: toward realizing people-centered care and outcomes management. J Acad Nutr Diet.

[45] Brickley B, Williams LT, Morgan M (2021). Putting patients first: development of a patient advocate and general practitioner-informed model of patient-centred care. BMC Health Serv Res.

[46] Ball L, Desbrow B, Leveritt M (2014). An exploration of individuals’ preferences for nutrition care from Australian primary care health professionals. Aust J Prim Health.

[47] Adamski M, Gibson S, Leech M, Truby H (2018). Are doctors nutritionists? What is the role of doctors in providing nutrition advice?. Nutr Bull.

[48] Ross LJ, Barnes KA, Ball LE (2019). Effectiveness of dietetic consultation for lowering blood lipid levels in the management of cardiovascular disease risk: a systematic review and meta-analysis of randomised controlled trials. Nutr Diet.

[49] Williams LT, Barnes K, Ball L (2019). How effective are dietitians in weight management? A systematic review and meta-analysis of randomized controlled trials. Healthcare (Basel).

